# Beyond the Headache: A Subtle Horner’s Syndrome Revealing Carotid Artery Dissection

**DOI:** 10.7759/cureus.96747

**Published:** 2025-11-13

**Authors:** Saravanaa Sankar, Taofeek Ojewuyi, Alekya Siddabathula, Naveeda Mahar, Pavankumar Narayanan, Mohammed Ali

**Affiliations:** 1 Internal Medicine/Acute Medicine, Southend University Hospital NHS Foundation Trust, Southend-on-Sea, GBR; 2 Diabetes and Endocrinology, Mid and South Essex NHS Foundation Trust, Southend-on-Sea, GBR; 3 Emergency Medicine, Mercy Hospital St. Louis, St. Louis, USA; 4 Acute Medicine, Southend University Hospital, Mid and South Essex NHS Foundation Trust, Southend-on-Sea, GBR

**Keywords:** carotid dissections, horners syndrome, internal carotid artery (ica), non-traumatic carotid artery dissection, stroke

## Abstract

Partial Horner’s syndrome, characterized by ptosis and miosis without anhidrosis, is a classic but often underrecognized manifestation of internal carotid artery (ICA) dissection and may represent the sole clinical indicator of an evolving vascular pathology.

Horner’s syndrome is a neurological disorder caused by interruption of the sympathetic nerve pathways that supply the eye and face. Despite its clearly defined clinical features, Horner’s syndrome is relatively uncommon, and standardized management guidelines are limited. Early detection relies on CT angiography (CTA), as the initial non-contrast CT can often be unremarkable.

We report a 60-year-old male who presented with headache and features of partial Horner’s syndrome, subsequently diagnosed with ICA dissection. This case highlights the need for clinicians to maintain a high index of suspicion for cervical artery dissection in patients presenting with unexplained Horner’s syndrome, even in the absence of trauma or neurological deficit. Early recognition and urgent vascular imaging are essential for accurate diagnosis and prevention of potentially life-threatening complications.

## Introduction

Cervical internal carotid artery (ICA) dissection is an important cause of ischemic stroke, particularly among younger individuals, though it can also occur in older adults and may present with subtle or atypical symptoms [1]. Horner’s syndrome refers to a collection of symptoms that occur when the sympathetic nervous pathway is disrupted. Clinically, it presents with ipsilateral miosis (pupil constriction), ptosis (drooping of the upper eyelid), and occasionally facial anhidrosis (loss of sweating). These features result from unopposed parasympathetic activity due to loss of sympathetic tone. Miosis occurs because of dominance of the iris constrictor muscle; ptosis results from involvement of the superior tarsal (Müller’s) muscle; and anhidrosis arises from sympathetic denervation of the facial sweat glands, though this is less frequent in postganglionic lesions [2].

The sympathetic pathway to the eye has three parts. The first-order neuron originates in the hypothalamus and travels down through the brainstem to the ciliospinal center of Budge (C8-T2). The second-order neuron exits the spinal cord, passes near the lung apex, and terminates in the superior cervical ganglion, making it vulnerable to injury from neck or upper chest surgery or a Pancoast tumor. The third-order neuron follows the ICA into the cavernous sinus, joins the ophthalmic branch of the trigeminal nerve, and reaches the iris dilator and Müller’s muscles, controlling pupil dilation and eyelid elevation [3].

Disruption at any point along this pathway produces Horner’s syndrome, and the clinical presentation varies depending on the lesion site. The underlying causes range from benign to potentially serious conditions, including infections, tumors, and vascular disorders such as carotid artery dissection. Therefore, early recognition and accurate localization of the lesion are essential for appropriate management and prevention of complications.

## Case presentation

A 60-year-old man with a history of poorly controlled hypertension presented with a two-day history of a persistent, dull headache localized to the left side. The headache was unrelieved by over-the-counter analgesics and was not associated with nausea, vomiting, photophobia, or phonophobia. There was no recent history of trauma, chiropractic manipulation, or infection. On arrival, his blood pressure was markedly elevated at 220/140 mmHg, and he admitted to poor adherence to antihypertensive therapy prescribed following his diagnosis of hypertension one year earlier.

**Figure 1:**
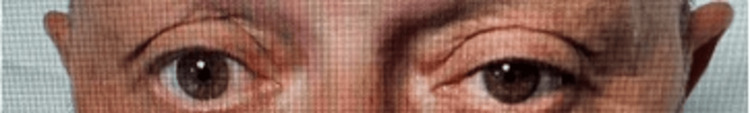
Partial Horner’s syndrome. Miosis and ptosis in the left eye.

Neurological examination revealed features consistent with partial left-sided Horner’s syndrome, characterized by ipsilateral ptosis and miosis (Figure 1). The left pupil measured 2 mm and was reactive to light, while the right pupil measured 5 mm and was equally reactive. Extraocular movements, facial symmetry, limb strength, coordination, and sensory function were normal, with no evidence of anhidrosis or cerebellar dysfunction.

A non-contrast CT scan of the brain was unremarkable, effectively excluding acute intracranial hemorrhage or mass effect. However, due to the presence of painful partial Horner’s syndrome, carotid artery dissection was suspected. The patient’s blood pressure was carefully managed using a titrated intravenous glyceryl trinitrate (GTN) infusion to maintain systolic blood pressure within the target range, minimizing the risk of propagation of the dissection while ensuring adequate cerebral perfusion.

**Figure 2:**
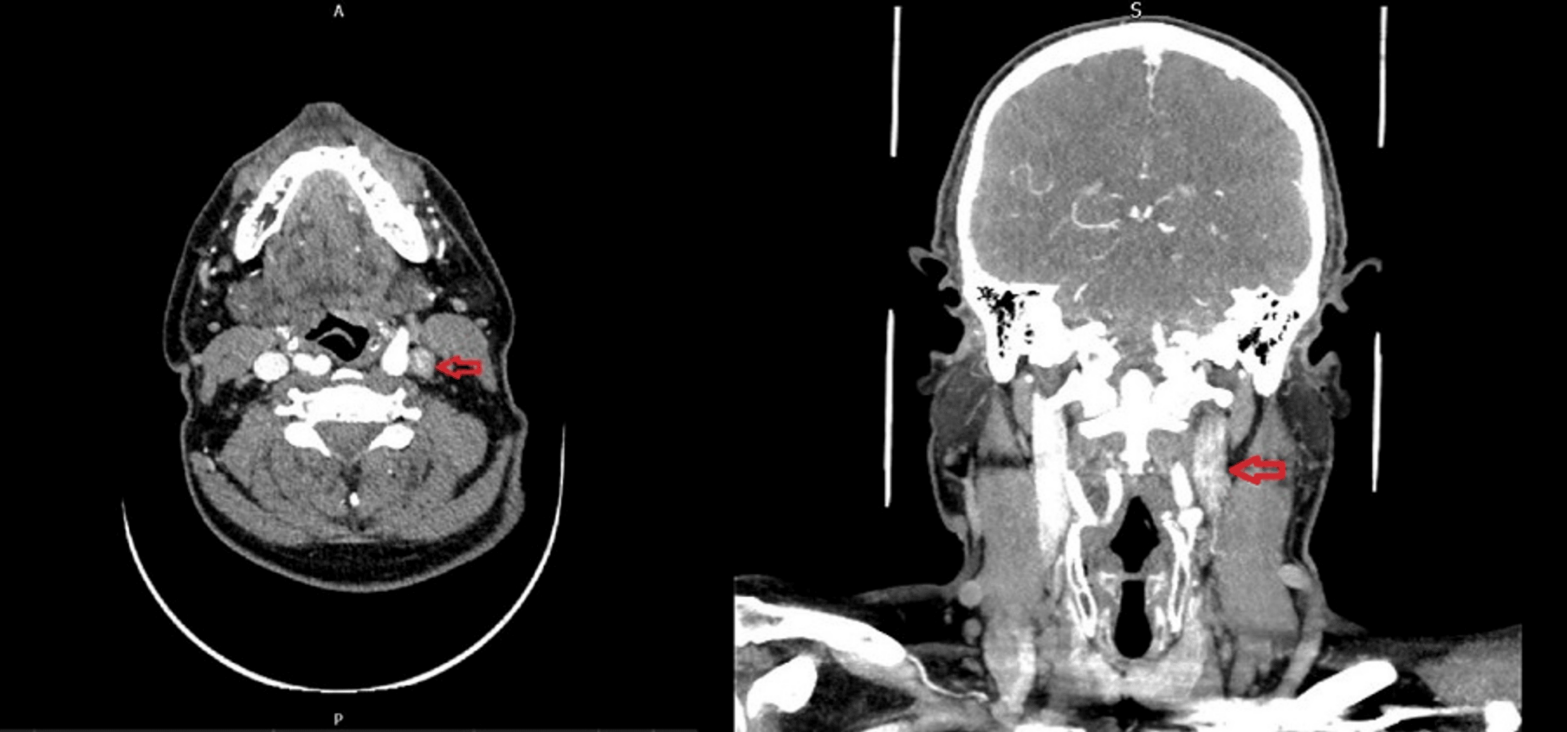
CT angiogram. Axial and coronal CT images demonstrating circumferential narrowing of the left internal carotid artery (ICA).

Subsequent CT angiography (CTA) of the head and neck demonstrated a segmental, tapered narrowing in the distal 15 mm of the left cervical ICA, consistent with arterial dissection (Figure 2). The patient was started on antiplatelet therapy following current recommendations for extracranial carotid artery dissection [4]. He was referred to a neurovascular specialist, who recommended a conservative approach with follow-up MRI to confirm the diagnosis and monitor progress.

## Discussion

This case illustrates a subtle presentation of ICA dissection in an older adult. While more common in younger patients, spontaneous dissections can occur at any age. An intramural hematoma can compress sympathetic fibers along the cervical ICA, resulting in partial Horner’s syndrome (ptosis and miosis without anhidrosis) [5]. The absence of other neurological deficits can delay diagnosis, highlighting the need for vigilance in patients presenting with isolated ptosis, miosis, or atypical headaches [6].

Imaging is critical for diagnosis. CTA is the initial modality of choice, revealing features such as tapering (“string sign”), double lumen, or pseudoaneurysm. MRI with fat saturation is the gold standard for detecting intramural hematoma, even when CTA findings are subtle.

Management focuses on reducing thromboembolic risk. Early recognition allows prompt initiation of dual antiplatelet therapy (aspirin and clopidogrel), as supported by the Cervical Artery Dissection in Stroke Study (CADISS) trial, which demonstrated comparable efficacy to anticoagulation in preventing recurrent events [7].

A multidisciplinary approach ensures optimal care: neurologists assess neurological deficits and initiate imaging; radiologists interpret subtle vascular findings; the stroke team guides secondary prevention; and vascular surgeons intervene when medical management is insufficient or symptoms persist. Dual antithrombotic therapy is generally continued for 3-6 months, with extension beyond six months if follow-up CT angiography shows persistent stenosis, occlusion, or pseudoaneurysm.

This case emphasizes that ICA dissection should be considered in isolated partial Horner’s syndrome, that symptom improvement does not rule out serious pathology, and that timely imaging and coordinated care are essential to prevent cerebrovascular complications [8].

## Conclusions

This case underscores the importance of recognizing partial Horner’s syndrome as a potential early indicator of ICA dissection. A high index of suspicion and timely vascular imaging are vital to prevent missed diagnoses and reduce the risk of stroke. Early initiation of antithrombotic therapy can mitigate ischemic complications, while strict blood pressure control and adherence to antihypertensive treatment are crucial, particularly in patients with known hypertension who are noncompliant with their medications, to prevent further vascular events.
